# Stimulating myocardial pyruvate dehydrogenase activity fails to alleviate cardiac abnormalities in a mouse model of human Barth syndrome

**DOI:** 10.3389/fcvm.2022.997352

**Published:** 2022-09-23

**Authors:** Amanda A. Greenwell, Seyed Amirhossein Tabatabaei Dakhili, Keshav Gopal, Christina T. Saed, Jordan S. F. Chan, Nick Kazungu Mugabo, Pavel Zhabyeyev, Farah Eaton, Jennifer Kruger, Gavin Y. Oudit, John R. Ussher

**Affiliations:** ^1^Faculty of Pharmacy and Pharmaceutical Sciences, University of Alberta, Edmonton, AB, Canada; ^2^Cardiovascular Research Centre, University of Alberta, Edmonton, AB, Canada; ^3^Women and Children's Health Research Institute, University of Alberta, Edmonton, AB, Canada; ^4^Division of Cardiology, Department of Medicine, University of Alberta, Edmonton, AB, Canada; ^5^Mazankowski Alberta Heart Institute, University of Alberta, Edmonton, AB, Canada; ^6^Health Sciences Laboratory Animal Services, University of Alberta, Edmonton, AB, Canada

**Keywords:** dichloroacetate, Barth syndrome, cardiomyopathy, pyruvate dehydrogenase, glucose oxidation

## Abstract

Barth syndrome (BTHS) is a rare genetic disorder due to mutations in the *TAFAZZIN* gene, leading to impaired maturation of cardiolipin and thereby adversely affecting mitochondrial function and energy metabolism, often resulting in cardiomyopathy. In a murine model of BTHS involving short-hairpin RNA mediated knockdown of *Tafazzin* (TazKD mice), myocardial glucose oxidation rates were markedly reduced, likely secondary to an impairment in the activity of pyruvate dehydrogenase (PDH), the rate-limiting enzyme of glucose oxidation. Furthermore, TazKD mice exhibited cardiac hypertrophy with minimal cardiac dysfunction. Because the stimulation of myocardial glucose oxidation has been shown to alleviate diabetic cardiomyopathy and heart failure, we hypothesized that stimulating PDH activity would alleviate the cardiac hypertrophy present in TazKD mice. In order to address our hypothesis, 6-week-old male TazKD mice and their wild-type (WT) littermates were treated with dichloroacetate (DCA; 70 mM in the drinking water), which stimulates PDH activity via inhibiting PDH kinase to prevent inhibitory phosphorylation of PDH. We utilized ultrasound echocardiography to assess cardiac function and left ventricular wall structure in all mice prior to and following 6-weeks of treatment. Consistent with systemic activation of PDH and glucose oxidation, DCA treatment improved glycemia in both TazKD mice and their WT littermates, and decreased PDH phosphorylation equivalently at all 3 of its inhibitory sites (serine 293/300/232). However, DCA treatment had no impact on left ventricular structure, or systolic and diastolic function in TazKD mice. Therefore, it is unlikely that stimulating glucose oxidation is a viable target to improve BTHS-related cardiomyopathy.

## Introduction

Severe, infantile-onset cardiomyopathy, often leading to heart failure, is the dominant clinical manifestation of the rare genetic disorder, Barth syndrome (BTHS) (1, 2). Pathogenic variants of *TAFAZZIN* on chromosome Xq28.12 cause BTHS by impairing the enzymatic activity of tafazzin, leading to defective cardiolipin (CL) remodeling and consequent mitochondrial abnormalities (3). Similar to other cardiac pathologies (4, 5), deficient myocardial energy production is a characteristic feature of BTHS-related cardiomyopathy (6, 7). However, the specific mechanisms by which tafazzin deficiency precipitates a cardiac energy deficit and whether optimization of oxidative metabolism represents a potential therapeutic target in BTHS requires further investigation.

Although disruption of tafazzin mediated CL remodeling is associated with electron transport chain (ETC) dysfunction (8–10), evidence has identified that derangements in oxidative metabolism may be substrate-specific, thus implicating upstream intermediary metabolism pathway defects in the pathogenesis of BTHS-related cardiomyopathy (11–13). Notably, studies utilizing the *Tafazzin* knockdown (TazKD) mouse model and *Tafazzin* knockout cell lines have identified a selective defect in the activity of pyruvate dehydrogenase (PDH), the rate-limiting enzyme of glucose oxidation (10, 13). Furthermore, glucose oxidation rates are markedly impaired in perfused isolated working hearts from TazKD mice (13).

Dichloroacetate (DCA) is a pyruvate analog that enhances PDH activity and glucose oxidation by inhibiting all isoforms of PDH kinase (PDHK), which inhibit PDH through reversible phosphorylation (14, 15). Of interest, stimulating myocardial PDH activity and subsequent glucose oxidation has been shown to improve cardiac function in murine models of diabetic cardiomyopathy, ischemia-reperfusion injury, and heart failure (16–19). We hypothesized that this may also represent a novel approach for the treatment of BTHS-related cardiomyopathy, of which no specific therapies have been identified to date. Accordingly, in the present study we aimed to determine whether enhancement of myocardial PDH activity via treatment with DCA would be effective in attenuating the development and progression of pathological cardiac structural remodeling in TazKD mice.

## Methods

### Animal care and experimentation

All animal procedures were approved by the University of Alberta Health Sciences Animal Welfare Committee and performed in accordance with the regulations of the Canadian Council on Animal Care. Animals were housed in a 22°C temperature-controlled unit under a 12-h light/dark cycle with standard environmental enrichment and *ad libitum* access to drinking water and food. The generation of the doxycycline-inducible short hairpin RNA (shRNA)-mediated TazKD mouse model has been described elsewhere (20). Doxycycline (625 mg/kg) was administered as part of the chow provided to the mice throughout the study. Female C57BL/6J mice were placed on doxycycline-containing diet 1-week prior to mating with transgenic male mice heterozygous for the *Tafazzin* shRNA transgene, to produce a *Tafazzin* deficiency in the pups during the early embryonic stage. Male littermates that did not possess the *Tafazzin* shRNA transgene were also maintained on the doxycycline-containing chow and used as our wild-type (WT) controls. Male TazKD and WT mice at 6-weeks of age were randomized to receive a 6-week treatment with sodium DCA (Sigma-Aldrich) which was added to the drinking water (70 mM) and pH-balanced. Daily water consumption for each cage of mice was measured consistently throughout the 6-week treatment period and calculated per mouse by dividing the total amount consumed by the number of mice in each cage. Blood glucose levels were measured from tail whole-blood during the random-fed state using the Contour Next blood glucose monitoring system (Bayer, NJ, USA). At the end of the treatment protocol, mice were euthanized with an intraperitoneal injection of sodium pentobarbital (12 mg) after a 16-h fast and 4-h refeed period. Peripheral tissues were subsequently extracted and immediately snap frozen in liquid nitrogen using liquid nitrogen-cooled Wollenberger tongs prior to storage at −80°C.

### Ultrasound echocardiography

Cardiac ultrasound images were acquired utilizing an MX 550S probe and the VisualSonics Vevo 3100 rodent ultrasound imaging system as previously described (13, 19). Mice were initially anesthetized with 2–3% isoflurane and maintained on 1–1.5% isoflurane for the remainder of the assessment. Body temperature, respiratory rate and heart rate were consistently monitored during image acquisition. Cardiac structure and left ventricular (LV) systolic and diastolic function were determined in 5-week-old WT and TazKD mice (baseline), and following 6-weeks of DCA treatment. Several parameters were assessed including, but not limited to, anterior and posterior wall dimensions, LV ejection fraction (EF), LV fractional shortening (FS), cardiac output (CO), mitral E/A ratio, tissue Doppler e′/a′ ratio, and the E/e′ ratio as described previously (13, 19). Because the E and e′ waves can become fused with A and a′ waves at higher heart rates, respectively, the E/A, E/e′ and e′/a′ ratios could not be accurately measured for some mice (21).

### Magnetic resonance imaging

Quantitative nuclear magnetic resonance relaxometry utilizing an EchoMRI-4in1/700 body composition analyzer was used to quantify total fat and lean mass as previously described (22).

### Western blotting

A protein lysis buffer containing 50 mM Tris HCl (pH 8 at 4°C), 1 mM EDTA, 10% glycerol (w/v), 0.02% Brij-35 (w/v), 1 mM dithiothreitol, and protease and phosphatase inhibitors (Sigma-Aldrich) was used to extract protein from powdered, frozen cardiac tissue (~15–20 mg). Protein was quantified using a Bradford protein assay kit (Bio-Rad) and samples (30 μg) were subsequently denatured and subjected to western blotting protocols as previously described (22). Membranes were probed with the following antibodies: PDH (3205S, Cell Signaling), phospho-PDH-E1α (Serine 293) (AP1062, Sigma-Aldrich), phospho-PDH-E1α (Serine 232) (AP1063, Sigma-Aldrich), phospho-PDH-E1α (Serine 300) (AP1064, Sigma-Aldrich), PDHK4 (ab214938, Abcam), vinculin (1390S, Cell Signaling), All antibodies were prepared in a 1/1000 dilution in 3% BSA except for vinculin, which was prepared in a 1/2000 dilution.

### Real-time quantitative PCR

First-strand cDNA was synthesized from RNA extracted from powdered frozen cardiac (~15–20 mg) tissue using the iScript Reverse Transcription Supermix (Bio-Rad Laboratories Inc., Hercules, CA). A CFX Connect Real-Time PCR machine (Bio-Rad Laboratories Inc.) was used to perform real-time PCR utilizing SYBR Green (KK4601; Kapa Viosystems, Inc.). Cyclophilin A (*Ppia*) was used as an internal housekeeping gene to determine relative mRNA transcript levels quantified with the 2^−ΔΔCt^ method as previously described (23). Primer sequences used include the following: *Ppia* forward; GCTGGACCAAACACAAACG, *Ppia* reverse; ATGCCTTCTTTCACCTTCCC, *Acta1* forward; CGACGGGCAGGTCATCA, *Acta1* reverse; ACCGATAAAGGAAGGCTGGAA, *Nppb* forward; GAGGTCACTCCTATCCTCTGG, *Nppb* reverse; GCCATTTCCTCCGACTTTTCTC.

### Protein carbonylation

Myocardial protein carbonylation was determined using the protein carbonyl content assay kit (MAK094, Sigma-Aldrich). Approximately 20 mg of frozen heart tissue per sample was lysed with protein lysis buffer and the resulting supernatant was analyzed according to the manufacturer's protocol, with absorbance measured at λ = 375 nm using a Synergy H1 microplate reader (BioTek). Protein carbonyl levels in myocardial samples were expressed as nmol/mg protein.

### Statistical analysis

All values are presented as mean ± standard error of the mean (SEM). An unpaired, two-tailed Student's *t* test or a two-way ANOVA was used to assess statistical significance and differences were considered significant when *P* < 0.05. Statistical analysis was completed utilizing GraphPad Prism 9 software.

## Results

### TazKD mice present with cardiac hypertrophy at 5-weeks of age

**Figure 1:**
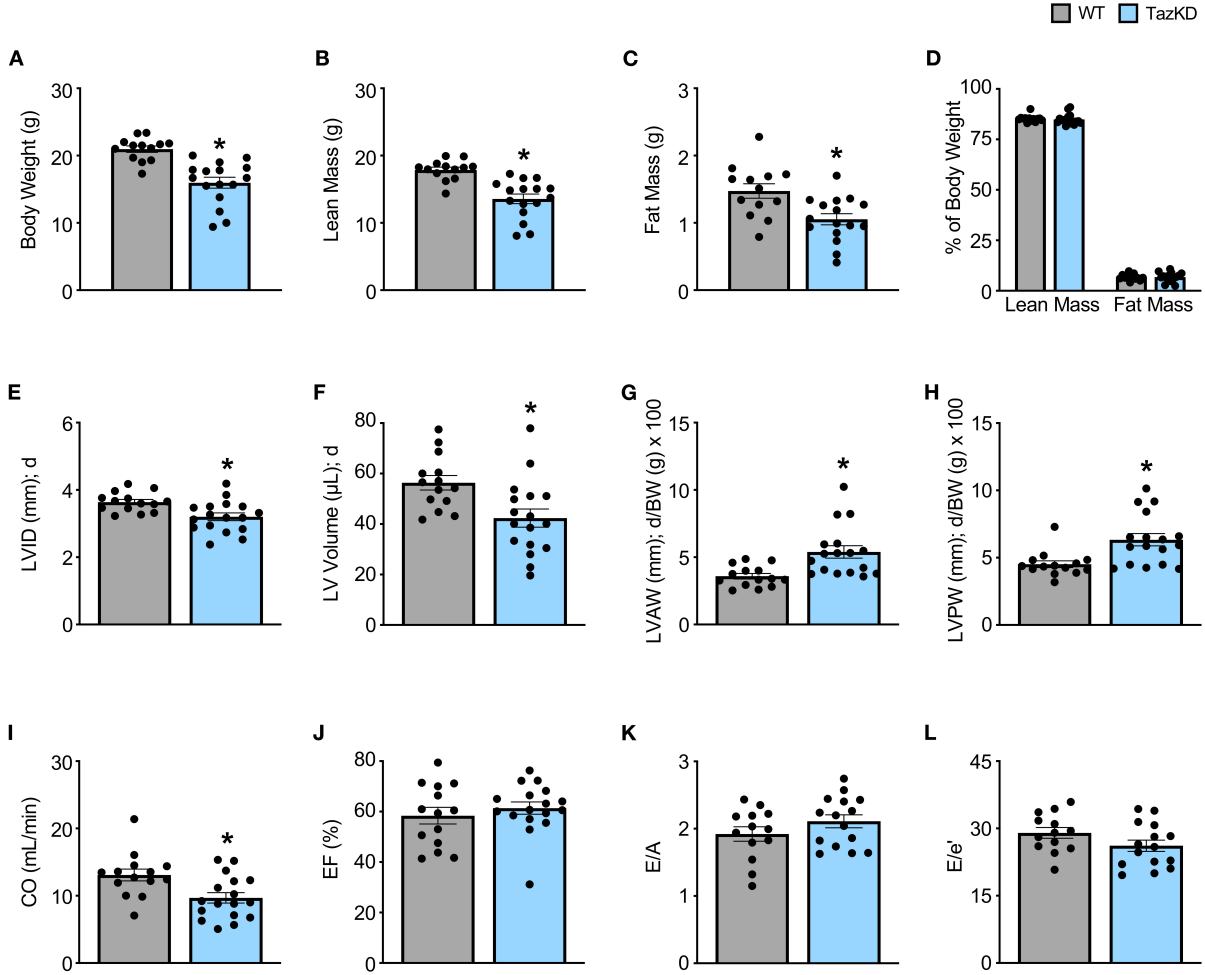
Cardiac hypertrophy is already present in TazKD mice at 5 weeks of age. **(A–D)** Body weight and body composition (lean mass, fat mass) analysis in 5-week-old TazKD mice and their WT littermates (WT: *n* = 13, TazKD: *n* = 16). Ultrasound echocardiography was used to assess left ventricular (LV) chamber and wall dimensions including **(E)** LV internal diameter (LVID), **(F)** LV volume, **(G)** LV anterior wall thickness (LVAW), **(H)** and LV posterior wall thickness (LVPW) during diastole (d), in addition to functional parameters including **(I)** cardiac output (CO), **(J)** ejection fraction (EF), **(K)** E/A, and **(L)** E/e' in 5-week old TazKD mice and their WT littermates. For structural and systolic function parameters (WT: *n* = 14, TazKD: *n* = 17); for diastolic function parameters (WT: *n* = 13, TazKD: *n* = 15). Values represent mean ± SEM. Differences were determined using an unpaired, two-tailed Student's *t* test. *P < 0.05 significantly different from WT littermates. BW, body weight.

5-week old TazKD mice displayed significant reductions in body weight, lean mass, and fat mass in comparison to their age-matched WT littermates (Figures 1A–C). Consistent with the generalized growth defect observed in subjects with BTHS (24), despite lean and fat mass being reduced in TazKD mice, the relative proportions of lean and fat mass normalized to body weight were similar between TazKD mice and their WT littermates (Figure 1D). As our previous findings identified that TazKD mice demonstrate cardiac hypertrophy by 8–10 weeks of age (13), we first performed ultrasound echocardiography studies in 5-week-old TazKD mice to determine whether this cardiac hypertrophy was present earlier. Despite LV mass being similar in 5-week-old TazKD mice and their WT littermates, when taking the generalized growth defect into account and normalizing LV mass to body weight, the TazKD mice demonstrated a clear cardiac hypertrophy (4.02 ± 0.12 mg/g [WT] vs. 4.65 ± 0.18 mg/g [TazKD]; *P* = 0.01). Furthermore, other indices of cardiac hypertrophy were evident in TazKD mice, including a decreased LV internal diameter (LVID) and volumes during diastole, as well as an increased LV anterior wall (LVAW) and posterior wall (LVPW) thickness when normalized to body weight (Figures 1E–H, Table 1). These structural abnormalities resulted in a 26% and 22% reduction in CO and stroke volume (SV), respectively, which is consistent with previous reports that TazKD mice do exhibit cardiac hypertrophy (Figure 1I, Table 1) (25). Parameters of systolic function including LVEF and LVFS were not different between TazKD and WT mice (Figure 1J, Table 1). Likewise, the E/A and E/e′ ratios were also similar between TazKD mice and their WT littermates, indicating normal diastolic function (Figures 1K,L, Table 1).

**Table 1:** *In vivo* baseline assessment of cardiac structural and functional parameters in WT and TazKD mice.

	**WT**	**TazKD**
Heart rate (beats/min)	401 ± 9	382 ± 6
BW (g)	19.8 ± 0.8	14.7 ± 0.9^*^
LVID (mm); s	2.53 ± 0.11	2.18 ± 0.11^*^
LVID (mm); d	3.64 ± 0.08	3.20 ± 0.11^*^
Volume (μL); s	24.0 ± 2.6	17.1 ± 2.6
Volume (μL); d	56.4 ± 2.9	42.4 ± 3.6^*^
Stroke Volume (μL)	32.4 ± 1.9	25.3 ± 1.9^*^
EF (%)	58.4 ± 3.3	61.4 ± 2.4
FS (%)	30.9 ± 2.3	32.3 ± 1.6
CO (ml • min^−1^)	13.1 ± 0.9	9.7 ± 0.8^*^
LVAW/BW × 10^2^ (mm/g); s	5.44 ± 0.30	7.57 ± 0.59^*^
LVAW/BW × 10^2^ (mm/g); d	3.59 ± 0.21	5.39 ± 0.46^*^
LVPW/BW × 10^2^ (mm/g); s	5.89 ± 0.26	8.15 ± 0.58^*^
LVPW/BW × 10^2^ (mm/g); d	4.52 ± 0.25	6.33 ± 0.46^*^
E/A	1.92 ± 0.11	2.11 ± 0.10
e′/a′	1.54 ± 0.10	2.09 ± 0.16^*^
E/e′	29.0 ± 1.2	26.1 ± 1.3

*In vivo* cardiac function and LV wall measurements in 5-week-old WT and TazKD mice (n = 13-17). Values represent mean ± SEM.

* P < 0.05, significantly different from WT.

BW, body weight; CO, cardiac output; EF, ejection fraction; FS, fractional shortening; LV, left ventricular; LVAW, LV anterior wall thickness; LVID, LV internal diameter; LVPW, LV posterior wall thickness; s, systole; d, diastole.

### Treatment with DCA decreases myocardial PDH phosphorylation in TazKD mice

**Figure 2:**
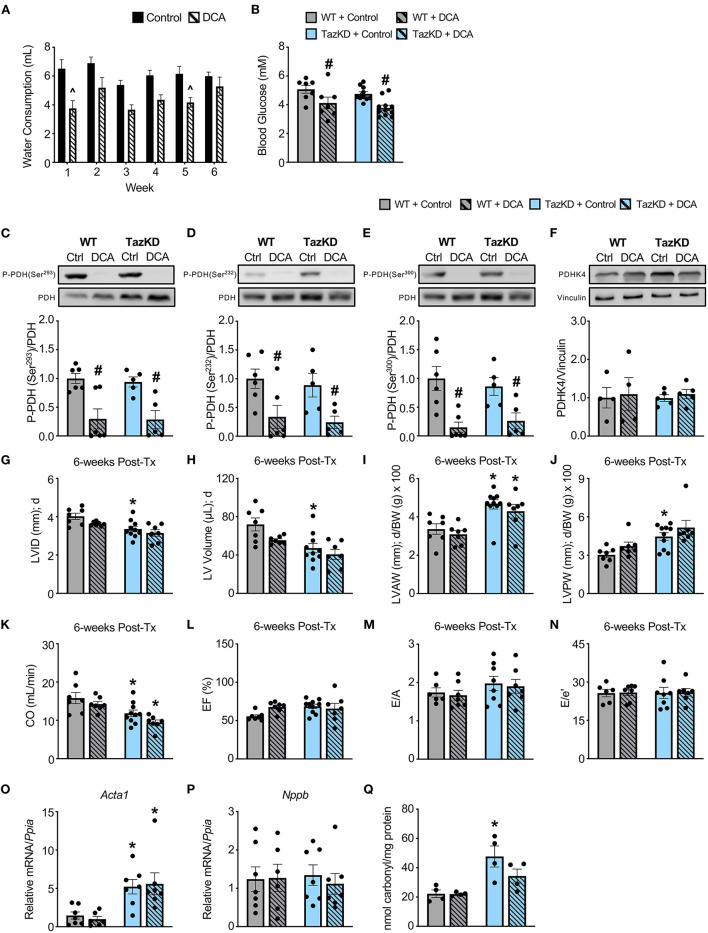
DCA treatment decreases inhibitory PDH phosphorylation but fails to alleviate the cardiac structural abnormalities in TazKD mice. **(A)** Average daily consumption of water or water supplemented with DCA (70 mM) reported per individual mouse for each week of the experimental protocol (*n* = 17–18). **(B)** Random-fed blood glucose measurements of WT and TazKD mice either subjected to control or DCA treatment (*n* = 7–11). Pyruvate dehydrogenase (PDH) phosphorylation at **(C)** serine 293, **(D)** serine 232, and **(E)** serine 300 relative to total PDH in myocardial tissue from WT and TazKD mice treated with control or DCA for 6-weeks (*n* = 5–6). **(F)** PDHK4 protein expression relative to vinculin in myocardial tissue from WT and TazKD mice treated with control or DCA for 6-weeks (*n* = 4–5). Ultrasound echocardiography was used to assess left ventricular (LV) chamber and wall dimensions including **(G)** LV internal diameter (LVID), **(H)** LV volume, **(I)** LV anterior wall thickness (LVAW), and **(J)** LV posterior wall thickness (LVPW) during diastole (d), in addition to functional parameters including **(K)** cardiac output (CO), **(L)** ejection fraction (EF), **(M)** E/A, and **(N)** E/e' in WT and TazKD mice treated with control of DCA. For structural and systolic function parameters (WT control: *n* = 7, WT DCA: *n* = 7, TazKD control: *n* = 10, TazKD DCA: *n* = 7); for diastolic function parameters (WT control: *n* = 6, WT DCA: *n* = 7, TazKD control: *n* = 8, TazKD DCA: *n* = 7). Relative mRNA expression of **(O)** skeletal α-actin (*Acta1*) and **(P)** brain natriuretic peptide (*Nppb*) normalized to cyclophilin A (*Ppia*), and **(Q)** total protein carbonylation in myocardial tissue from WT and TazKD mice treated with control or DCA for 6-weeks (*n* = 4-7). Values represent mean ± SEM. Differences were determined using a two-way ANOVA. ^∧^*P* < 0.05, significantly different from control treated mice. ^#^*P* < 0.05, significantly different from control treated counterpart. *P < 0.05, significantly different from WT counterpart. BW, body weight.

In order to correct the impairments in myocardial PDH activity we previously observed in TazKD mice (13), we treated 6-week-old TazKD mice and their WT littermates with the PDHK inhibitor, DCA (70 mM in the drinking water), for 6-weeks. Although WT and TazKD mice treated with DCA via the drinking water tended to consume slightly less water than control mice on average, water consumption was similar across the 6-week treatment period, thus ensuring that the dose of DCA each mouse received remained consistent throughout the study (Figure 2A). In line with a systemic enhancement of glucose oxidation, blood glucose levels were significantly decreased in both WT and TazKD mice treated with DCA (Figure 2B). As glucose is a more oxygen efficient fuel than fat (26), stimulating PDH activity often leads to a reduction in oxygen consumption, and we also observed with indirect calorimetry that DCA treatment decreased whole-body oxygen consumption rates in both WT and TazKD mice (*data not shown*). Furthermore, we observed a marked reduction in the inhibitory phosphorylation of PDH at all three phosphorylation sites (Serine 293, Serine 232, and Serine 300) in myocardial tissue from DCA-treated WT and TazKD mice without altering PDHK4 protein expression (Figures 2C–F). Taken together, these findings are highly suggestive of an increase in myocardial PDH activity and a subsequent enhancement of myocardial glucose oxidation.

### Treatment with DCA does not improve the cardiac structural abnormalities in TazKD mice

In opposition of our hypothesis, the adverse hypertrophic cardiac remodeling present in TazKD mice was not improved by treatment with DCA. The decreased LVID and LV volume during systole and diastole in TazKD mice were unaffected by DCA treatment (Figures 2G,H, Table 2). Furthermore, the increased LVAW and LVPW thickness present in TazKD mice were also not improved following treatment with DCA (Figures 2I,J). Consistent with unattenuated cardiac hypertrophy, CO and SV remained decreased in DCA treated TazKD mice (Figure 2K, Table 2). Systolic function represented by LVEF and LVFS, as well as diastolic function represented by the E/A, e′/a′, and E/e′, were similar between TazKD mice and their WT littermates, while being unaffected via DCA treatment (Figures 2L–N, Table 2).

**Table 2:** *In vivo* assessment of cardiac structural and functional parameters in WT and TazKD mice following treatment with DCA.

	**WT**	**WT**	**TazKD**	**TazKD**
	**Control**	**DCA**	**Control**	**DCA**
Heart rate (beats/min)	400 ± 14	378 ± 8	377 ± 11	379 ± 8
BW (g)	26.0 ± 0.7	22.6 ± 1.0^#^	21.2 ± 0.7^*^	20.0 ± 0.9
LVID (mm); s	2.87 ± 0.15	2.28 ± 0.08	2.11 ± 0.15^*^	2.02 ± 0.28
LVID (mm); d	4.02 ± 0.17	3.63 ± 0.04	3.36 ± 0.14^*^	3.15 ± 0.18
Volume (μL); s	32.4 ± 4.0	17.9 ± 1.6^#^	15.8 ± 3.0^*^	15.9 ± 4.4
Volume (μL); d	71.9 ± 6.8	55.6 ± 1.5	47.3 ± 4.9^*^	40.7 ± 5.2
Stroke Volume (μL)	39.5 ± 3.2	37.7 ± 1.5	31.5 ± 2.3	24.9 ± 1.7^*^
EF (%)	55.5 ± 2.1	67.8 ± 2.5	68.6 ± 2.8	65.7 ± 6.7
FS (%)	28.7 ± 1.4	37.3 ± 1.9	37.9 ± 2.1	37.5 ± 6.1
CO (ml • min^−1^)	15.8 ± 1.5	14.2 ± 0.7	11.8 ± 0.9^*^	9.44 ± 0.7^*^
LVAW/BW × 10^2^ (mm/g); s	4.82 ± 0.30	5.02 ± 0.41	6.79 ± 0.29^*^	6.36 ± 0.49
LVAW/BW × 10^2^ (mm/g); d	3.36 ± 0.28	3.09 ± 0.19	4.66 ± 0.25^*^	4.30 ± 0.35^*^
LVPW/BW × 10^2^ (mm/g); s	4.19 ± 0.31	5.49 ± 0.39	6.27 ± 0.39^*^	6.65 ± 0.50
LVPW/BW × 10^2^ (mm/g); d	3.03 ± 0.22	3.72 ± 0.31	4.46 ± 0.30^*^	5.16 ± 0.55^∧^
E/A	1.74 ± 0.12	1.67 ± 0.13	1.98 ± 0.19	1.90 ± 0.18
e′/a′	1.40 ± 0.13	1.56 ± 0.11	1.86 ± 0.15	1.90 ± 0.18
E/e′	25.7 ± 1.5	25.9 ± 1.2	25.7 ± 2.2	26.0 ± 1.5

*In vivo* cardiac function and LV wall measurements in WT and TazKD mice subjected to control or DCA treatment for 6-weeks (n = 6–10). Values represent mean ± SEM.

* P < 0.05, significantly different from WT counterpart.

# P < 0.05, significantly different from control treated counterpart.

∧ P = 0.07, vs. WT counterpart. BW, body weight; CO, cardiac output; EF, ejection fraction; FS, fractional shortening; LV, left ventricular; LVAW, LV anterior wall thickness; LVID, LV internal diameter; LVPW, LV posterior wall thickness; s, systole; d, diastole.

We next assessed markers of cardiac remodeling and cardiac dysfunction, whereby mRNA expression of skeletal α-actin (*Acta1*), a marker of cardiomyocyte hypertrophy, remained markedly elevated in myocardial tissue from TazKD mice and was unaffected by DCA treatment (Figure 2O). In addition, myocardial mRNA expression of brain natriuretic peptide (*Nppb*) was not different between WT and TazKD mice, nor was its expression affected by DCA treatment (Figure 2P). As TazKD mice exhibit increases in myocardial oxidative stress (25), we also assessed myocardial protein carbonylation levels, which were increased in control treated TazKD mice vs. their WT littermates but prevented via treatment with DCA (Figure 2Q).

## Discussion

Perturbations in myocardial energy metabolism play a significant role in the development and progression of numerous cardiovascular disorders, and therefore represent a promising target for therapeutic intervention (27, 28). This study sought to elucidate whether optimization of cardiac glucose oxidation, through treatment with DCA to stimulate PDH activity, could improve the pathological structural remodeling of the heart associated with *Tafazzin* deficiency observed in BTHS. Our results demonstrated that treatment of TazKD mice with DCA for 6-weeks did not attenuate or reverse their cardiac hypertrophy despite a restoration of myocardial PDH activity.

We were particularly surprised by these negative findings, since stimulating myocardial PDH activity has been shown to improve cardiac abnormalities in numerous experimental settings. For example, inhibition of the transcription factor forkhead box O1 prevents transcription of *Pdk4*, which encodes for PDHK4, thereby increasing myocardial glucose oxidation and alleviating the diastolic dysfunction associated with experimental diabetic cardiomyopathy in mice (19). Similarly, the glucagon-like peptide-1 receptor agonist liraglutide, an antidiabetic agent that promotes insulin secretion, also stimulates myocardial PDH activity and glucose oxidation, thereby alleviating the diastolic dysfunction in a murine model of diabetic cardiomyopathy (29). Treatment with DCA itself has also been shown to alleviate experimental diabetic cardiomyopathy in rats (18), while decreasing infarct size in mice subjected to experimental ischemia-reperfusion injury via temporary occlusion of the left anterior descending coronary artery (17). In addition, DCA treatment of Dahl salt-sensitive rats fed a high-salt diet increased myocardial PDH activity, which attenuated heart failure progression as indicated by increased systolic function, decreased cardiac hypertrophy, and improved survival (16).

Because isolated working hearts from TazKD mice exhibit marked reductions in glucose oxidation rates (13), it seemed well rationalized to presume that DCA treatment might alleviate their cardiac abnormalities. This raises the important question as to why stimulating myocardial PDH activity would fail to yield benefit in TazKD mice, considering numerous other cardiac pathologies are corrected by such a metabolic strategy? We posit that the persistent destabilization of ETC supercomplexes and reduced ETC complex activity coupled with consequent elevations of mitochondrial reactive oxygen species and oxidative stress (10, 11, 30–32), likely supersede intermediary metabolism defects present in BTHS. This may explain the failure of DCA treatment to attenuate the LV hypertrophic remodeling in TazKD mice despite an improvement of myocardial glucose oxidation. Therefore, the results of our study suggest that any therapeutic intervention aiming to optimize myocardial intermediary energy metabolism may be ineffective at alleviating BTHS-related cardiomyopathy, unless the defects in the respiratory chain are addressed. In other words, even if the BTHS heart is capable of oxidizing more of the carbohydrate or fatty acid fuel delivered to it, if the generated reducing equivalents (i.e. NADH) are unable to result in ATP generation due to the ETC respiratory defects, no improvement in cardiac pathology can be expected. However, our preliminary observations addressing this question suggest that DCA can still increase myocardial ATP levels in TazKD mice (control treated mean of 2.53 mM [n = 2] vs. DCA treated mean of 4.00 mM [n = 3]), and thus we plan to interrogate this more extensively in future studies.

In order for metabolic interventions to yield clinical utility in BTHS, it may need to be coupled with strategies that ultimately correct the adverse CL remodeling responsible for BTHS-related ETC respiratory dysfunction. One such strategy that may show promise is the agent, elamipretide, which is a cell-permeable, aromatic-cationic mitochondria-targeting tetrapeptide that localizes to the inner mitochondrial membrane where it selectively associates with CL to improve respiratory chain function (33, 34). Presently, the ongoing TAZPOWER clinical trial (NCT03098797) is investigating the efficacy of directly targeting mitochondrial dysfunction with elamipretide as a treatment for BTHS (35). Intriguingly, in participants with genetically confirmed BTHS, a 16% improvement in average SV indexed to body surface area at week 36 of the open extension phase of the trial compared to baseline was reported with elamipretide treatment (mean age 19.5 years, range 12–35 years). Furthermore, elamipretide treatment produced a trend toward an increase in SV over time, when a slope model of individual regression lines for each subject was utilized (35). It is possible that the utilization of a metabolic therapy such as DCA in combination with elamipretide may ensure that augmented substrate oxidation maximally translates to enhanced ATP production. This may thus represent a more effective approach to mitigate BTHS-related cardiomyopathy, which we emphasize should be a key direction for metabolic investigations to reorient their research toward.

An earlier intervention with DCA may also need to be considered, since we already observed cardiac hypertrophy in TazKD mice at 5-weeks of age. Thus, administering DCA immediately after birth or the weaning of TazKD mice may allow us to prevent the progression of their cardiac hypertrophy before it develops. Alternatively, it may be that glucose oxidation is simply not a viable metabolic target for BTHS-related cardiomyopathy, but not myocardial intermediary metabolism *per se*. Indeed, increasing fatty acid oxidation by treatment of 3-month-old TazKD mice with a pan-peroxisome proliferator activated receptor (PPAR) agonist, bezafibrate, prevented the development of dilated cardiomyopathy and systolic dysfunction over the course of 4-months (36, 37). A complementary study also determined that bezafibrate treatment prevented the exacerbation of cardiac dysfunction in 4.5-month-old TazKD mice in combination with infusion of isoproterenol (38). Bezafibrate is also presently being investigated as a potential therapy for BTHS in the CARDIOlipin MANipulation (CARDIOMAN) trial (39). Therefore, interrogation of other intermediary metabolism pathways rather than glucose oxidation may be a more beneficial target. However, bezafibrate treatment also induced an increase in mitochondrial biogenesis, the enzymatic activity of ETC complexes I-III and trended to increase the protein expression of all ETC complexes in the hearts of TazKD mice (38). A limitation of our study is that we did not assess ETC function or other parameters of mitochondrial status in response to DCA treatment (e.g., were defects in complex I-V activity in TazKD mice mitigated by DCA?). Conversely, it has also been demonstrated that fibrates actually decrease myocardial fatty acid oxidation, since PPAR activation in the liver increases fatty acid oxidation, which decreases hepatic triacylglycerol secretion and subsequent fatty acid delivery to the myocardium (40). As such, it is difficult to determine the specific mechanism(s) underpinning the cardioprotective actions of bezafibrate in BTHS.

In summary, the findings of the present study reveal that treatment with DCA is ineffective in reversing the cardiac hypertrophy present in TazKD mice, and thus may not represent an effective treatment for BTHS-related cardiomyopathy. Although a precise mechanism to explain why DCA failed to alleviate cardiac remodeling in the *Tafazzin* deficient heart is unknown, persistent respiratory chain dysfunction may have limited energy production despite an enhancement of flux through PDH. As such, further investigation is required to determine whether DCA in combination with a therapy that stabilizes the ETC, such as elamipretide, may represent a beneficial therapeutic approach for BTHS-related cardiomyopathy.

## Data availability statement

The raw data supporting the conclusions of this article will be made available by the authors on reasonable request.

## Ethics statement

The animal study was reviewed and approved by the University of Alberta Health Sciences Animal Welfare Committee.

## Author contributions

AG, ST, and JU conceived and designed research. AG, ST, KG, CS, JC, NK, PZ, FE, and JK performed experiments and analyzed data. AG and JU drafted the manuscript. AG, ST, GO, and JU edited and revised the manuscript. JU approved final version of the manuscript and takes full responsibility for the data within this paper. All authors contributed to the article and approved the submitted version.

## Conflict of interest

The authors declare that the research was conducted in the absence of any commercial or financial relationships that could be construed as a potential conflict of interest.

## Publisher's note

All claims expressed in this article are solely those of the authors and do not necessarily represent those of their affiliated organizations, or those of the publisher, the editors and the reviewers. Any product that may be evaluated in this article, or claim that may be made by its manufacturer, is not guaranteed or endorsed by the publisher.
